# Fatal outcome of malignant transformation of hidradenitis suppurativa: A case report and literature review

**DOI:** 10.1002/ccr3.2608

**Published:** 2020-02-06

**Authors:** Valdemar Wendelboe Nielsen, Astrid‐Helene Ravn Jørgensen, Simon Francis Thomsen

**Affiliations:** ^1^ Department of Dermato‐Venereology & Wound Healing Centre Bispebjerg Hospital Copenhagen Denmark; ^2^ Department of Biomedical Sciences University of Copenhagen Copenhagen Denmark

**Keywords:** chronic inflammation, hidradenitis suppurativa, squamous cell carcinoma, vulvar cancer

## Abstract

Squamous cell carcinoma arising in hidradenitis suppurativa (HS) is a rare albeit the most serious complication in HS, with a reported fatality of up to 42.9%. This calls for greater clinical awareness in patients with long‐standing chronic HS.

## Graphical abstract

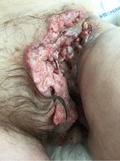

## 1 INTRODUCTION

We present a case of a 66‐year‐old woman with metastatic vulvar squamous cell carcinoma that developed within an area of chronic hidradenitis suppurativa in the groin and review the literature. The progression of SCC was so severe that she passed away only a month into her hospital stay.

Hidradenitis suppurativa (HS) is a chronic inflammatory skin disorder, characterized by recurrent deep‐seated, inflammatory nodules in apocrine gland–bearing areas of the body, that is, the axillae, groin, and perineum. The nodules may progressively expand to abscesses and draining sinus tracts with subsequent scarring. Chronic, long‐standing inflammation in HS may lead to the development of squamous cell carcinoma (SCC), a rare albeit the most serious complication of HS.

We present a case herein of a woman who developed SCC of the vulva in the setting of chronic, long‐standing HS with fatal outcome and update the literature on this topic to date.

A search in the PubMed database was conducted until the 25th of September 2019 to identify articles describing patients with HS who developed SCC. All of the articles reviewed were published in English. The search terminology included the following: “Hidradenitis suppurativa AND squamous cell carcinoma.”

## 2 CASE REPORT

The patient was a 66‐year‐old Caucasian female with a history of HS since 2010 with Hurley stage III lesions in the groins with six nodules and six sinus tracts. She had a HSS score of 63, DLQI score of 13, and visual analogue scale (VAS) for overall disease‐related distress of 5.5 and VAS for boil‐associated pain from boils in the past month was 0. The patient also had hypertension, smoked 20 cigarettes per day for 54 years and had a BMI of 26.4 kg/m^2^. She had no family history of HS. The patient was first seen in our clinic in 2016, where treatment with topical clindamycin twice daily and oral tetracycline 500 mg twice daily was initiated along with topical corticosteroids for local eczema. At 3‐month follow‐up, the skin was characterized by fibrotic and thickened cicatricial areas with purulent drainage and inflamed nodules. Topical metronidazole and oral lymecycline 300 mg twice daily were administered and the patient was referred to wide excisional plastic surgery of the groins, provided she stopped smoking 2 months prior to surgery.

**Figure 1:**
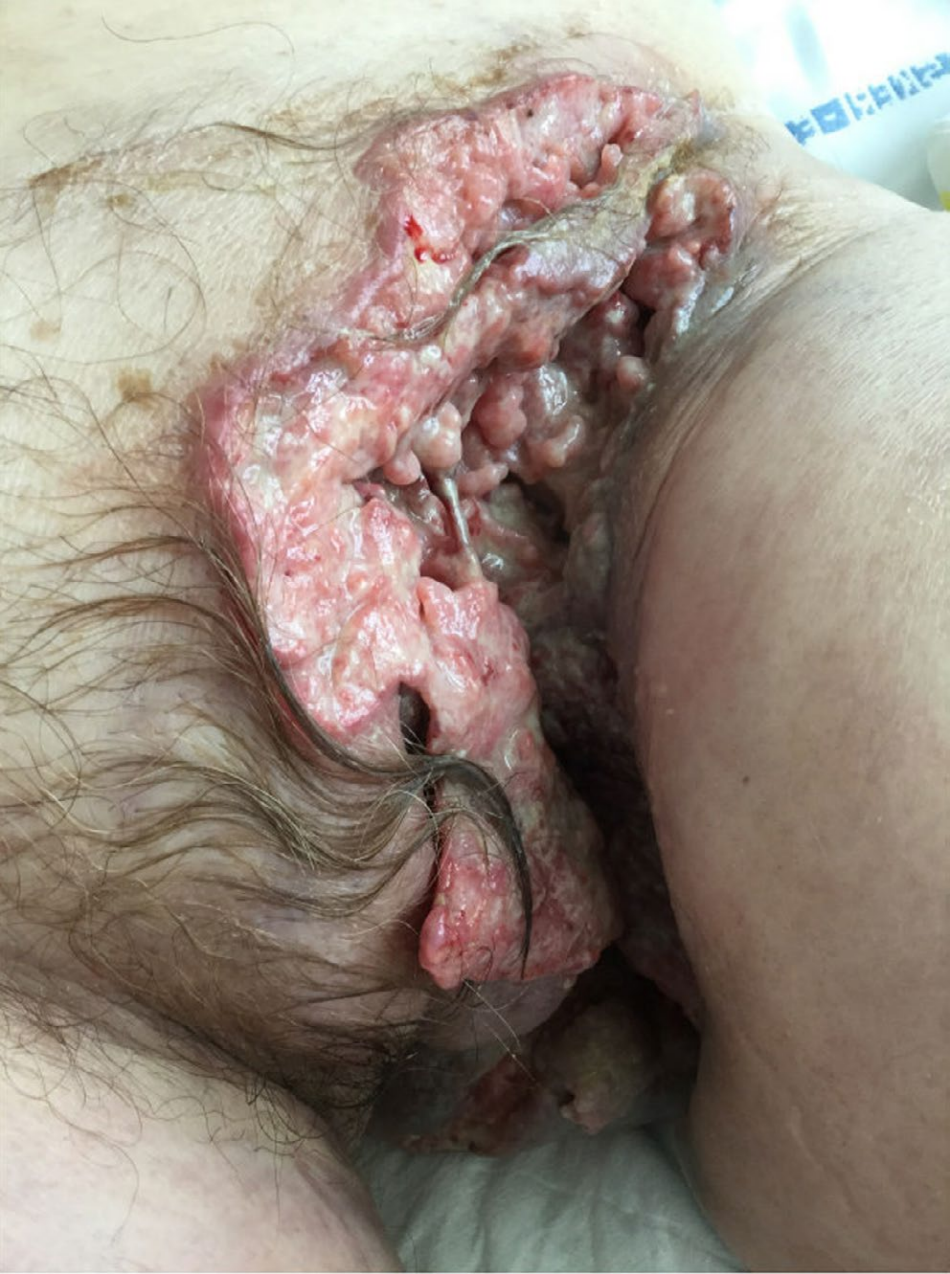
Squamous cell carcinoma arising in hidradenitis suppurativa in left groin. Presented with multiple severe fistulae in skin and subcutis, a large ulcerating cancer with drainage and necrosis of lymph nodes

Surgery was canceled due to continued smoking, and no further surgery was planned. She failed to seek further help for her HS until she was admitted 3 years later after severe worsening of HS, with multiple severe fistulae and a large foul‐smelling tumor with drainage in the left groin and necrosis of lymph nodes (Figure 1). A PET‐CT showed ingrowth and destruction of the left ramus inferior of the pelvis and gland metastasis under the diaphragm. Biopsy demonstrated invasive poorly differentiated SCC, transformed from several years of chronic HS. The clinical picture was compatible with ulcerating vulvar cancer, and the patient received radiation therapy as a palliative measure. However, she was shortly thereafter deemed terminal and died 1 month after the initial metastatic SCC diagnosis.

### 2.1 Prevalence of squamous cell carcinoma in patients with hidradenitis suppurativa

A paper in 2016 by Jourabchi et al^1^ and in 2018 by Chapman et al^2^ described 80 and five cases, respectively, on SCC in patients with HS cases in the English literature. We found five additional cases of SCC arising from HS reported in the literature since the last updated review by Chapman et al^2^ (Table 1). Among these five new cases, the mean history of HS prior to SCC diagnosis was 16 years, and the mean age at diagnosis was 51.2 years. SCC developed predominantly from long‐standing HS of the gluteal and perianal areas. A history of tobacco use was reported in two of the five cases. Interestingly, the occurrence of SCC in the context of axillary HS was not reported in the English literature before Dessinioti.^3^

**Table 1:** Update of published patients with squamous cell carcinoma arising in hidradenitis suppurativa reported since Chapman et al in 2018^2^

Author	Year	Age (y)	Sex (M/F)	Duration of HS prior to SCC (years)	Location of SCC	Metastasis (Y/N)	HPV status	Tobacco use (Y/N)	Outcome
Harview et al^9^	2018	47	M	10	Perineal	N	Positive	Unknown	Recurrent SCC in a perineal polypoid growth 2 mo into his hospital stay. Alive and undergoing chemotherapy at the time of publication.
Miura et al^10^	2018	45	M	20	Buttock	Y	Negative	Y	Started radiotherapy, but died of multiple organ failure 4 mo later
Zhang et al^11^	2017	59	M	29	Buttock	Y	Unknown	Y	Died 5 mo after metastatic SCC diagnosis
McArdle et al^12^	2017	49	F	13	Sacral‐buttock/ perianal	Unknown	Unknown	Unknown	A wide local excision was performed and was diagnosed with ulcerative colitis several years later
Dessinioti et al^3^	2017	56	M	8	Left axillae	N	Unknown	Unknown	Complete surgical excision of the lesion
Own patient	2019	66	F	9	Left groin/ vulvar	Y	Unknown	Y	Died one month after metastatic SCC diagnosis

Abbreviations: F, female; HS, hidradenitis suppurativa; M, male; N, no; SCC, squamous cell carcinoma; Y, yes.

## 3 DISCUSSION

Our case highlights a rare consequence of HS, where long‐standing and poorly controlled inflammation transforms into SCC. The development of SCC in the setting of HS is thought to be rare, with an estimated prevalence of SCC among patients with HS of up to 4.6%, concluded in a cohort study of 217 patients with HS by Lavogiez et al^4^ The prognosis is poor due to the advanced stage of SCC at the time of diagnosis and difficulty in obtaining a representative biopsy in early stages of cancer development. A review of published cases of SCC arising in chronic HS determined that nine of the 21 patients (42.9%) died of their disease.^5^ Including our patient, three of three of the six patients (50%) in this review passed away after their metastatic SCC diagnosis, supporting the severity of this compilation. However, fatality could be speculated to be overemphasized due to publication bias.

The diagnosis of malignancy in patients with HS is less straight forward, since chronic draining wounds can mimic malignant ulceration. Biopsies are not required to diagnose HS, but should be considered in the setting of an uncertain clinical picture to rule out malignant transformation.

The Danish Board of Health recommends a 6‐week smoking cessation prior to surgical removal of HS. However, our patient continued smoking and was withheld surgical excision while her HS severely worsened, to a point where surgery was not possible anymore. It is an ethical dilemma, whether surgery should be postponed until smoking cessation in every case. In a former study by Losanoff,^6^ surgery is the only known treatment method that provides a real chance for a cure for both HS and a complicating carcinoma. Patients that do not initially undergo surgery for severe HS can potentially develop cancerous degeneration as in the present case report. This is why complete excision in severe cases of HS is also indicated to avoid chronic progression of the disease. Retrospectively, less strict smoking policy could have allowed surgery and perhaps avoided cancer transformation and death. It is important to consider the socioeconomic situation of the patient in the treatment as well. Our patient only had one distant relative for support, lived alone and could not take care of herself, so she failed to seek medical attention even though her disease severely worsened. Support for the patients with low socioeconomic status including smoking cessation and weight loss should be considered for optimal wound healing^7^ and adherence to therapy.

Biologic drugs are used for moderate to severe HS or in nonoperable patients, and have shown promising results.^8^ However, they are speculated to be associated with increased risk of nonmelanoma skin cancer due to their immunosuppressive properties. Further, it is well known that chronic skin inflammation is linked to higher risk of malignant transformation as our case supports. Further research focusing on the risk of developing SCC in HS patients is needed to elucidate whether long‐term treatment with biologics may lead to prevention or promotion of malignant transformation of chronic skin inflammation. Our patient did not receive biologic therapy.

## 4 CONCLUSION

In conclusion, patients suffering from chronic inflammatory processes should be closely monitored with a high index of suspicion of malignancy, since SCC in long‐standing HS is a rare, but potentially fatal complication. HS in the perianal, perineal, and sacral region should be treated early with wide and radical surgical excision due to the risk of malignant degeneration and development of aggressive SCC particularly in these regions. Dispensation from smoking policy should be considered in selected patients.

## CONFLICT OF INTEREST

The authors have nothing to declare.

## AUTHOR CONTRIBUTIONS

Valdemar Wendelboe Nielsen: performed literature review, collected the data, analyzed the data, and prepared and revised the manuscript. Astrid‐Helene Ravn Jørgensen: substantially contributed and provided inputs to the content, performed literature review, and critically revised the manuscript. Simon Francis Thomsen involved in idea and data collection and critically revised the manuscript.
